# A new complex mental health test in a positive psychological framework

**DOI:** 10.3389/fpsyg.2022.775622

**Published:** 2022-09-02

**Authors:** Virág Zábó, Attila Oláh, András Vargha

**Affiliations:** ^1^Doctoral School of Psychology, ELTE Eötvös Loránd University, Budapest, Hungary; ^2^Institute of Psychology, ELTE Eötvös Loránd University, Budapest, Hungary; ^3^Institute of Research on Adult Education and Knowledge Management, ELTE Eötvös Loránd University, Budapest, Hungary; ^4^Person- and Family-oriented Health Science Research Group, Institute of Psychology, Faculty of Humanities and Social Sciences, Károli Gáspár University of the Reformed Church in Hungary, Budapest, Hungary

**Keywords:** happiness, subjective well-being, mental health, mental health test, positive psychology (PP1.0 and PP2.0), positive psychological assessments, maintainable positive mental health theory

## Abstract

According to the Maintainable Positive Mental Health Theory (MPMHT), the main pillars of positive mental health are global well-being, efficient coping that enables an individual to maintain positive conditions and functioning, savoring capacity, resilience, and dynamic self-regulation. This study presents the validation of a new five-scale mental health test (MHT), the MHT that operationalizes MPMHT. The methodology comprised two online cross-sectional studies using self-report questionnaires. Participants in Study I (*n* = 1,736; 448 males, 1,288 females; mean age 51.3 years; SD = 11.6 years) filled in the MHT, the Flow, the Positive emotions, Engagement, Positive Relationship, Meaning, Accomplishment Questionnaire (PERMA-Profiler), and the Flourishing Scale. Participants in Study II (*n* = 1,083; 233 males, 847 females; mean age 33.9 years; *SD* = 12.2 years) filled in the MHT, the Shortened Aspiration Index, the short form of the Beck Depression Inventory, the WHO Well-Being Index, the Satisfaction with Life Scale, the Purpose in Life Test, and the Schema Questionnaire–Short Form. Exploratory factor analysis (EFA) identified a five-factor structure with 17 items in Study I that was confirmed with excellent fit measures in confirmatory factor analysis in Study II. Both studies indicated a high level of internal consistency (above 0.70). In each subscale, a minimum part of 44% did not overlap with the set of the other subscales. The content validity of the subscales was confirmed by 10 tests of mental health. We found a positive correlation of the self-regulation and resilience subscales with age, while women showed a higher level of savoring than men at all age levels. When Study I was replicated after 2 weeks and again after 11 months, excellent internal consistency and good test–retest correlation values of the MHT scales were found. The MHT can thus be considered a reliable and valid measurement tool for mental health.

## Introduction

The present study proposes a new theory of mental health, the Maintainable Positive Mental Health Theory (MPMHT). First, we present the theoretical framework of our new positive mental health concept. In the next step, we provide an overview of the models of well-being and mental health to date, and we highlight the gaps between these and our proposed concept. By presenting the five pillars of MPMHT, we aim to shed light on how our new model integrates and complement the accumulated knowledge of the science of mental health measures to date.

### The conceptualization of maintainable positive mental health theory

A central topic in positive psychology is the identification of indicators (symptoms) of mental health and the elaboration of models to serve as a theoretical framework when developing a diagnostic system in the realm of positive mental health. Such a diagnostic system could provide a positive alternative to the *Diagnostic and Statistical Manual of Mental Disorders* (DSM), which has long been used in clinical practice. Given that, in identifying mental health, the absence of mental disease is not an adequate definition, it is necessary first to develop a positive concept of mental health.

We argue that the clarification of the concept of well-being, the theoretical and empirical analysis of the relationship between well-being and mental health, and the rehabilitation of classical interpretations of mental health can lead to an integrative concept of the positive realm of mental health. According to our MPMHT, the level of well-being will depend both on the presence or absence of the capacities and psychological resources needed to ensure positive mental health, and on the ability to use these capacities. Such a concept would treat all theoretically and empirically identified components of well-being as the set of symptoms of mental health that reflect the presence and proper functioning of the psychological capacities needed to ensure and maintain positive mental health.

This new approach is in line with the mental health definition of the WHO (Galderisi et al., 2015). The World Health Organization (WHO) defines mental health as “a dynamic state of internal equilibrium which enables individuals to use their abilities in harmony with universal values of society. Basic cognitive and social skills; ability to recognize, express and modulate one’s own emotions, as well as empathize with others; flexibility and ability to cope with adverse life events and function in social roles; and harmonious relationship between body and mind represent important components of mental health which contribute, to varying degrees, to the state of internal equilibrium” (Galderisi et al., 2015). In other words, the components of well-being are not the only agents that contribute to positive mental health; resilience, accommodation to changes, and the development of efficient coping capacities (savoring and the ability to establish positive states and handle negative states) are also major contributors.

Maintainable positive mental health theory endorses the view of what Keyes (2007; Keyes et al., 2020) emphasized, which is that mental disease and mental health are two separate dimensions that must be treated as independent continuums. At one pole of mental disease are intensive and frequent occurrences of mental disorders, while at the opposite pole are symptoms of mental disorders that are rarely present or that take insignificant forms. In the case of mental health, one pole represents the rare occurrence and weak appearance of mental health indicators, while the opposite pole represents the high frequency of positive mental health indicators.

### An overview of the concepts and the dimensions of previous models of well-being and mental health

Just as mental disorders comprise a theoretically based and empirically demonstrable set of symptoms, positive mental health can also be characterized by a clearly identifiable set of symptoms that can be framed within an appropriate theory and demonstrated and studied empirically.

#### Mental health as multidimensional well-being

According to one approach in positive psychology, mental health can essentially be defined according to the pillars of multidimensional well-being. Ryff’s (1995) multifactor psychological well-being scale integrates the six components of overall well-functioning but does not cover hedonic well-being which is also an important component of mental health. Keyes (2007) 13-dimensions mental health questionnaire does not comprehensively include all the important capabilities (e.g., savoring, self-regulation, resilience) and components (e.g., spiritual well-being) which are essential in achieving a high degree of positive mental health. Diener’s and his colleagues’ flourishing scale (Diener et al., 2009) only measures psychological well-being, SPANE (Diener et al., 2009) only assess emotions, and Positive emotions, Engagement, positive Relationship, Meaning, Accomplishment (PERMA) (Seligman, 2018) describes the factors of well-being, but none of them cover the competencies and abilities that play an irreplaceable role in mental health.

#### Mental health as mirror opposite to the symptoms of mental disorders

An alternative approach is to argue that mental health is characterized by symptoms that are the mirror opposite of mental disorders (Huppert and So, 2013; Caprara et al., 2019; Oláh, 2019). For example, the Positivity Scale (Caprara et al., 2019; Oláh, 2019) measures a combination of high self-esteem, life satisfaction, and optimism as suggested sources of a syndrome of optimal functioning. These models do not include any ability or capacity to guarantee mental health, only the resilience appears in Huppert and So’s (2013) flourishing questionnaire.

#### Mental health as flourishing

Although these approaches differ, 70% of their components, from which the multidimensional domain of mental health is constructed, are more or less identical. They share the common feature of integrating the hedonic and eudemonic approaches in the metaphoric concept of flourishing, which can be characterized by the simultaneous presence of positive feelings and good psychological functioning. Flourishing, as an umbrella concept for the components of well-being, is the positive pole or uppermost zone on the mental health continuum.

#### Mental health as “hedo-eudemonic” well-being

“Flourishing” is also used in the names of measurement tools that operationalize “hedo-eudemonic” models of well-being, for example Global Well-being Scale (Oláh, 2019; Oláh et al., 2020), Positive Mental Health Scale (Lukat et al., 2016) and Positive Functioning Inventory (Joseph and Maltby, 2014) capture only the hedonic and eudaimonic aspects of mental health. Measurement tools (or diagnostic scales) like Diener’s and his colleagues’ flourishing scale (Diener et al., 2009), SPANE (Diener et al., 2009) and a version of PERMA extended with overall well-being, negative emotions, loneliness, and physical health components (PERMA Profiler, Butler and Kern, 2016) encompass the major components of well-being and the symptoms of positive mental health in terms of their targets and measured content. Nevertheless, these hedo-eudemonic models do not cover all aspects of well-being (e.g., self-acceptance, spiritual well-being). In a recent review paper that outlined empirical studies covering 99 well-being measurement tools, 196 different components of well-being were identified (Linton et al., 2016).

#### Classical models of mental health

In the so-called classical models of mental health (Jahoda, 1958; Vaillant, 2003; Vaillant G. E., 2012) the focus is on personality traits that guarantee efficient self-regulation and flexible accommodation, and on psychological resources that foster fulfillment. For instance, the use of the character strengths that leads to human flourishing (Mayerson, 2020) are only predictors of the optimal mental health. The presence of personality traits resources is also regarded as a primary factor in positive mental health in salutogenic theory (Antonovsky, 1991), according to which mental health is maintained by efficient stress management, resilient self-regulation, and flexible accommodation to the continuously changing world (Block and Kremen, 1996; Block and Block, 2014). In theories of this kind, well-being is the consequence, or the result, of mental health and is due to resilient accommodation, capacities that establish a physical, psychological, and environmental balance, and well-functioning health maintenance skills. The PISI (Oláh, 2005) includes skills for self-efficacy and self-regulation, but does not cover global well-being, savoring, and resilience.

#### Balanced models of mental health

According to a recent model, the life balance and harmony model, the key to well-being is to maintain balance and harmony in all areas of human functioning (Lomas, 2021). In the equilibrium theory, a key factor in well-being is the maintenance of a relative balance between challenges and available resources, with an emphasis on the capacities needed to handle situations that are out of balance (Dodge et al., 2012). Apart from omitting some important elements of well-being, these models do not imply the abilities and capacities. Balance, harmony, and equilibrium could be a principle in the dynamic interaction of the pillars of mental health. Also, according to the capability theory of Amartya Sen (see, e.g., Walker and Unterhalter, 2007), capacities can explain the establishment and maintenance of all possible aspects of well-being. The focus of the model deviates from the essence of positive mental health and this approach can be a potential predictor of positive mental health. Sustainable Mental Health Model (Bohlmeijer and Westerhof, 2021) integrates dysfunctional and functional perspectives of mental health.

#### Mental health as the sum of the components of well-being

In earlier tools for the measurement of the realm of positive mental health (Bech et al., 1996; Diener et al., 2009; Lamers et al., 2011; Huppert and So, 2013; Butler and Kern, 2016; Lukat et al., 2016; Oláh et al., 2020), the focus has been primarily on the components of well-being, without taking into account all the aspects of mental health referred to in the WHO definition and in classical theories of mental health. Another feature of these tools is that they cannot be considered as the operationalization of a comprehensive mental health model. It is also important to note that the number of published measures of well-being and mental health has decreased significantly since 2010 (Lomas, 2021).

### Extracting the pillars of Maintainable Positive Mental Health Theory

The holistic concept of positive mental health must integrate the realm of well-being models, the psychological resources and capacities needed to ensure and maintain positive mental health, and the skills that guarantee their efficient functioning.

Our main goal is to develop a construct of mental health that differs both theoretically and empirically from other theoretically defined constructs of multidimensional well-being (flourishing, PERMA, etc.) called often as an equivalent or the top-zone of mental health. We assert that mental health is a broader concept than well-being, justified by research based on the classical theories of mental health (Antonovsky, 1991; Vaillant G., 2012) and on our own studies (Oláh, 2021; Nagy et al., 2021), which is also consistent with the mental health definition of the WHO (Galderisi et al., 2015). However, we do not encounter in the literature a measurement tool of mental health that could be considered an operationalizing construct of the WHO description of mental health, and we do not know also a mental health model that would integrate suggestions of scientific approaches for the pillars of positive mental health from classical models up to most recent theories of positive psychology. Keyes’ concept of mental health, the Flourishing construct of Diener and Huppert, and the PERMA model of Seligman all construe positive mental health based on well-being components. Building on classical and recent ideas of positive mental health, in the concept of Maintainable Positive Mental Health we would like to clarify that mental health is a function of individual capacities (resilience, creative and executive competencies) by means of which the individual can work up an equilibrium with the outside world, promoting his/her development, creating a steady state for within-person functioning (self-regulation), and an equilibrium of positive and negative emotions (coping, savoring). The existence and efficient functioning of these elements may lead to the global well-being, a multifaceted component of positive mental health. Summarizing, in our suggested definition mental health is a high level of global well-being which goes-together with psychological, social, and spiritual well-functioning, resilience, efficient creative and executive functioning, coping and savoring capacities, all pillars insuring the maintainability of mental health.

The first pillar in our new model is Global Well-being. MPMHT integrates existing well-being theories and the identified dimensions of well-being in the global well-being pillar. Global Well-being means multi-component subjective well-being encompassing emotional states and psychological functioning in the emotional, psychological, social, and spiritual areas in life (Oláh and Kapitány-Fövény, 2012; Oláh, 2016; Oláh et al., 2020). Table 1 indicates the pillars of Global-Well-being which goes hand in hand with savoring capacity, creative and executive efficiency, self-regulation, and resilience.

**TABLE 1:** Pillars of global well-being according to maintainable positive mental health theory.

Global well-being			
Emotional well-being	Positive functioning		Spiritual well-being
	Psychological well-being	Social well-being	
Positive affect Happiness Life Satisfaction	Self-acceptance Personal growth Environmental mastery Autonomy Positive relations with others	Social acceptance Social actualization Social contribution Social coherence Social integration	Joy of transcendence experience Joy of universality experience Vertical and horizontal responsibility

The second pillar is Savoring which refers to the ability and capacity which allows individuals to mentally mobilize their joyful memories and experiences to generate mental well-being, reliving them in the present and, furthermore, extending them to future events (Bryant and Veroff, 2007). Savoring is also a prerequisite for MPMHT because it is an ability that can guarantee the achievement and maintenance of positive mental health (Bryan et al., 2022).

Thirdly, utilizing Creative and Executive Efficiency competence, an individual becomes able to cope with the difficulties they encounter by mobilizing their various competencies in the difficult, stressful, and challenging situations of life. Furthermore, it indicates how individuals are able to provide successful individual and social problem-solving behavior (Oláh, 2005; Oláh et al., 2020).

Fourthly, the ability to regulate and control emotions, temperament, and negative states and to persist in achieving a given goal plays an important role in mental health (Oláh, 2005; Elliot et al., 2011; Singh and Sharma, 2018). Self-regulation is the capacity of the individual to disregard prominent responses and to regulate affects, cognitions, and behaviors. It is the ability to alter thoughts, feelings, desires, and actions in the perspective of such higher goals and would represent one of the most adaptive variables of human behavior (Vohs and Baumeister, 2004).

The fifth pillar is Resilience. With the ability of resilience, an individual is able to mobilize their mental capacities and resources to maintain positive mental health when they face unexpected, stressful, and difficult situations. The higher the level of resilience, the more quickly the individual is able to recover from a sudden, unexpected stressful situation (Connor and Davidson, 2003; Southwick and Charney, 2018; Verdolini et al., 2021). According to MPMHT, these independent components are together responsible for the mental health of the individual.

Although various psychological constructs exist, the mental health test (MHT) could be the first test to have a five-dimensional complex structure (Global Well-being, Savoring, Creative and Executive Efficiency, Self-regulation, and Resilience), with the aim of covering the wide spectrum of mental health. For these reasons, the present study aimed to operationalize the MPMHT by preparing the validation of the five-scale MHT on a Hungarian population.

## Overview of the present studies

Two studies were carried out to prepare and validate the final version of the MHT. The aim of Study I was to finalize with EFA a set of items for which the five-dimensional statistical model of the MHT yields appropriate fit indices. The main aim of Study II was to confirm the model on a new, independent sample. After this verification of structural validity, both studies were used to check the substantive validity of the MHT.

## Study I exploratory factor analysis

### Method

#### Participants and procedure

Participants completed a 64-item online questionnaire posted from mid-January 2020 for 2 months in Facebook groups that are frequently visited by adults of different ages^1^ and with different occupations and interests. Ethical approval for the study was granted by the Research Ethics Committee of the local university (permission number: 2019/61). Participation was voluntary and anonymous. Informed consent was obtained but no compensation was given. The valid study sample consisted of 1,736 adult persons (448 males and 1,288 females). Among them 1,540 persons were residents of Hungary, and 196 persons were residents of other countries who filled in the Hungarian-language online questionnaire^2^. The sociodemographic characteristics of the sample are summarized in Table 2.

**TABLE 2:** Sociodemographic characteristics of the participants of Study I (*n* = 1,736, 448 – 25.8% males, 1,288 – 74.2% females).

Age	18–25 years old: 1.9%	26–35 years old: 6.5%	36–50 years old: 38.9%	51–65 years old: 41.3%	66–90 years old: 11.4%
**Number of children**	0: 18.5%	1: 23.2%	2: 41.7%	3: 13.0%	3 + : 3.6%
**Type of city**	Village: 22.4%	Small town: 33.2%	Large town: 25.8%	Capital: 18.6%	
**Educational level**	Primary: 1.7%	Secondary: 38.2%	College: 35.2%	University: 24.9%	
**Marital status**	Lives alone: 28.9%	Civil partnership: 16.0%	Married: 50.1%	Widow: 5.0%	
**Profession**	Employee: 57.8%	Retired person: 20.7%	Entrepreneur: 14.5%	Unemployed: 3.9%	Other: 3.1%
**Financial status**	Poor: 2.0%	Below average: 5.3%	Average: 74.6%	Wealthy: 17.5%	Rich: 0.6%

Based on the data in Table 2, we can conclude that the Study I sample was sufficiently heterogeneous for us to draw valid conclusions about the MHT from the sample. Although the majority of participants in the sample were women (74.2%), the number of men (448) was also sufficiently large to ensure representative results.

In terms of age, the majority of the participants (38.9%) were middle-aged (36–50 years), although the number of people over 65 (198) was also substantial. There was a strikingly low proportion of young people aged between 18 and 25 (1.9%, 33 people). The sample was also balanced according to the type of city where participants lived, since the number of respondents in all categories was over 300. More than 98% of the participants had a high-school certificate. Regarding marital status, 50.1% of the participants were married, while a small proportion (5.0%) were widowed. The majority of the participants (57.8%) were employed, although there was also a significant proportion of retired participants (20.7%) and entrepreneurs (14.5%). The vast majority of participants in the sample (74.6%) considered their financial situation to be average, although there was also a non-negligible proportion of wealthy people (17.5%). Only a small proportion of respondents declared themselves to be poor (2.0%) or rich (0.6%).

#### Measures

Nine of the questions in the questionnaire referred to sociodemographic data (gender, age, place of residence, etc., see Table 2). One question (Positive experience%) assessed the proportion of the respondent’s recent positive experiences (1 = *10% positive experiences and 90% negative experiences* … 9 = *90% positive experiences and 10% negative experiences*). Four special questions, scored using a six-point Likert scale, assessed the physical and mental condition of the respondent: (1) Physical condition (My physical state is: 1 = *very bad*, 2 = *bad*, 3 = *acceptable*, 4 = *good*, 5 = *very good*, 6 = *excellent*); (2) General mental state (My general mental state is: 1 = *very bad*, 2 = *bad*, 3 = *acceptable*, 4 = *good*, 5 = *very good*, 6 = *excellent*); (3) General health condition (I am satisfied with my general health: 1 = *strongly disagree*, 2 = *moderately disagree*, 3 = *slightly disagree*, 4 = *slightly agree*, 5 = *moderately agree*, 6 = *strongly agree*); and (4) Physical strength (I feel strong and physically robust: 1 = *strongly disagree*, 2 = *moderately disagree*, 3 = *slightly disagree*, 4 = *slightly agree*, 5 = *moderately agree*, 6 = *strongly agree*).

##### Mental health test

The basic concept was to develop a mental health test (MHT) in the form of a short questionnaire comprising no more than 20 items to obtain a comprehensive picture of mental health according to MPMHT. The items on the five scales were selected based on the following arguments, considering that the item that most strongly represents the given measuring instrument should be selected (Oláh et al., 2018):

(1) Well-being. This scale is based on the following three areas of subjective well-being: (i) the ratio of positive and negative experiences (Fredrickson, 2009; Appendix, item 1); (ii) the subjective quality of the state of mental health and well-being (Lyubomirsky, 2010; Appendix, item 14); and (iii) the global level of happiness (Huppert and So, 2013; Appendix, item 18).
(2) Savoring. For this scale, three items were selected from the short Hungarian version of the Savoring Beliefs Inventory (Nagy et al., 2021) based on the criteria that they be comprehensive, simple, and highly representative of the total score of the questionnaire. The three items selected for the MHT according to these principles (Appendix, items 3, 10, and 12) explained the total score of the 10-item short Hungarian form of the Savoring Beliefs Inventory with a variance ratio of *R*^2^ = 0.81 based on annual data from the Happiness Map of Hungary between 2015 and 2019, with a sample size of 8,035^3^.
(3) Creative and Executive Efficiency. This scale consists of four items (Appendix, items 5, 7, 15, and 17) taken from the 16-item short version of the Psychological Immune Competence Inventory (PICI; see Oláh, 2005), using the same names. A fifth item, which was also considered an important component of creative and executive efficiency, was added from the 80-item version of the PICI (Oláh, 2005) (Appendix, item 9).
(4) Self-regulation. For this scale, three items were selected from the self-regulation subscale of the 16-item short version of the PICI (Oláh, 2005).
(5) Resilience. Items for this scale in the first version of the MHT (Oláh et al., 2018) were selected from the Resilience subscale of the 16-item short version of the PICI (Oláh, 2005). Taking into account the many different scales of resilience (Block and Kremen, 1996; Connor and Davidson, 2003; Smith et al., 2008; Windle et al., 2011), we were looking for one that is internationally recognized and that has already been translated into several languages. Applying linear regression analysis, four items (Appendix, items 4, 6, 11, and 13) of the six-item Brief Resilience Scale (Smith et al., 2008) explained the total score of the six-item test with a variance ratio of *R*^2^ = 0.95 on a sub-sample (*n* = 8,035) of the above-mentioned Happiness Map of Hungary.

Based on the above considerations, the MHT questionnaire comprises 18 items (see Appendix) divided into five scales of three, three, five, three, and four items respectively. The items are scored according to six-point Likert scales (1 = *does not agree at all*, 6 = *agrees completely*). The Well-being, Savoring, and Creative and Executive Efficiency subscales include positive items only. The Self-regulation subscale contains negative items only. The Resilience subscale consists of two positive and two negative items. The final scores are obtained by averaging the scores obtained for the items in each of the five scales.

##### Flow

The key element of Csíkszentmihályi’s flow construct (immersion in activity, constant interest) was examined based on Magyaródi et al. (2013, 2014), using the following item: “If something really interests me, I am able to do it with pleasure and in depth, even in difficult situations.”

##### Positive emotions, engagement, positive relationship, meaning, accomplishment questionnaire (PERMA-profiler)

The PERMA model was developed by Seligman (2018), building on his further developed earlier concept of authentic happiness. The components of the five-pillar model reinforce one another in creating and maintaining a state of well-being. The 23-item PERMA-Profiler (Butler and Kern, 2016) measures Seligman’s model using a 10-point Likert scale (0 = *never/not at all/terrible*; 10 = *always/completely/excellent*). In the case of 15 items, the five basic pillars are measured using three questions each; eight items, comprising three questions each, are used to assess negative emotions (e.g., “How often do you feel anxious?”) and health (e.g., “How would you rate your health?”). Among the 23 items one item is used to measure happiness (“All in all, how happy would you say you are?”) and one item is used to measure loneliness (“How lonely do you feel in your daily life?”). In terms of the scales that represent the five basic pillars, the Positive Emotion scale focuses on the frequency of experiencing positive emotions (e.g., “How often do you feel joyful?”). The Engagement scale refers to the frequency of experiencing flow and the absorption of cognitive and emotional resources (e.g., “How often do you become absorbed in what you are doing?”). The Meaning scale examines the tendency to set meaningful goals, seek meaning, and serve meaningful goals that transcend the self (e.g., “In general, to what extent do you lead a purposeful and meaningful life?”). The Accomplishment scale provides information about the extent to which one’s own successful performance and the joy of experiencing competence contribute to increasing well-being in an individual’s life (e.g., “How often do you achieve the important goals you have set for yourself?”). The Relationships scale measures the extent to which it is true for an individual’s life that happiness, love, and good relationships with others are the *sine qua non* of well-being (e.g., “To what extent do you feel loved?”). All the scales in the Hungarian version of the PERMA-Profiler (Oláh, 2016) showed reliable Cronbach’s alpha values: Positive Emotions: 0.88; Engagement: 0.57; Relationships: 0.79; Meaning: 0.76; Accomplishment: 0.74; Health: 0.88; and Negative Emotions: 0.77.

##### Flourishing scale

This eight-item scale (Diener et al., 2009) operationalizes an improved version of Diener’s concept of subjective well-being, in which, in addition to life satisfaction and the dominance of positive emotions, the necessity of competence, optimism, contributing to the well-being of others, life purpose, self-esteem, and positive relationships are highlighted. Cronbach’s alpha: 0.94.

To summarize, with one exception (the Engagement subscale of the PERMA-Profiler), the scales in the above tests all had excellent reliability, with Cronbach’s alpha values of above 0.74.

### Results

The internal consistency of all the scales in the MHT was computed with ROPstat (Vargha et al., 2015). Table 3 shows that the Cronbach’s alpha and McDonald’s omega values mostly being above 0.80 (DeVellis, 2016; Barbera et al., 2020) were adequate for all scales. One possible way to improve the internal consistency would be to drop one item from the Resilience subscale (item 6: “Stressful events are difficult to bear”). Calculating pairwise correlations of the scales, Table 4 shows that most of the scales have a moderate positive relationship with Pearson’s *r* of between 0.35 and 0.62 with two exceptions. This is consistent with the theory that different components of mental health are positively related to one another (Ryff and Marshall, 1999).

**TABLE 3:** Cronbach’s alpha and McDonald’s omega values measuring the internal consistency of the MHT scales in Study I.

Scale	Number of items	Study I (*n* = 1,736)	
		α	ω
Well-being	3	0.84	0.85
Savoring	3	0.85	0.85
Creative and Executive Efficiency	5	0.85	0.85
Self-regulation	3	0.85	0.85
Resilience	4	0.75	0.77
Resilience (without item 6)	3	0.74	0.78

α, Cronbach’s alpha; ω, McDonald’s omega.

**TABLE 4:** Intercorrelations and coefficients of the skewness and kurtosis of the MHT scales in Study I (*n* = 1,736).

Scale	Savoring	CEE	Self-regulation	Resilience	Skewness	Kurtosis
Well-being	0.62***	0.62***	0.35***	0.60***	–0.85***	0.61***
Savoring	1	0.61***	0.19***	0.46***	–0.79***	0.48***
Creative and Executive Efficiency		1	0.24***	0.49***	–0.78***	0.65***
Self-regulation			1	0.45***	–0.50***	–0.45***
Resilience				1	–0.34***	–0.01

***p < 0.001.

In the next step we performed a five-factor EFA using Mplus (Muthén and Muthén, 1998–2011) to verify that the 18 items of the MHT form five factors according to the constructed subscales. Due to the strongly non-normal distribution of the scales (see the last two columns of Table 4), a robust maximum likelihood estimator (MLR) (Maydeu-Olivares, 2017) was applied with Geomin oblique rotation, allowing correlating factors (Hattori et al., 2017). The rotated factor loading matrix is shown in Table 5. In this table, the item indices (i1, i2, etc.) correspond to the item indices in the Appendix, supplemented by the abbreviation of their scale (i1W, i2SR, etc.). Based on Table 5, we can conclude that each item in the Well-being, Savoring, Creative and Executive Efficiency and Self-regulation scales forms a separate factor with loadings above 0.60, with two exceptions (i12S: 0.40; i9C: 0.41). In the case of the Resilience scale, one of the four items loaded to Self-regulation scale (i6R: 0.50) and another item (i13R) loaded to the Self-regulation scale as well with a 0.38 loading (see Table 3).

**TABLE 5:** Five-factor exploratory factor analysis of the 18 items of the MHT using MLR method and Geomin oblique rotation on the data from Study I (*n* = 1,736): Five-factor factor weight matrix.

Item	Factor1	Factor2	Factor3	Factor4	Factor5
i1W	**0.67**	0.06	–0.01	0.03	–0.01
i14W	**0.85**	–0.01	0.03	0.01	0.04
i18W	**0.79**	0.00	–0.01	0.02	0.03
i3S	0.04	**0.72**	0.04	0.01	0.08
i10S	–0.02	**0.99**	–0.03	0.00	–0.04
i12S	0.32	**0.40**	0.09	0.01	0.14
i5C	–0.03	0.01	**0.78**	0.01	0.08
i7C	–0.03	0.00	**0.78**	0.04	0.04
i9C	0.30	0.14	**0.41**	–0.02	0.04
i15C	0.23	–0.06	**0.65**	0.03	–0.03
i17C	0.12	0.08	**0.51**	–0.06	–0.08
i2SR	0.02	0.07	0.01	**0.84**	–0.02
i8SR	0.08	0.03	0.00	**0.78**	–0.08
i16SR	–0.07	0.00	0.044	**0.75**	0.21
i4R	0.06	0.08	0.19	0.00	0.67
i6R	0.08	–0.07	–0.01	0.50	0.25
i11R	0.15	0.065	0.03	–0.01	**0.69**
i13R	–0.02	–0.046	–0.14	0.38	**0.50**

W, Well-being; S, Savoring; C, Creative and Executive Efficiency; SR, Self-regulation; R, Resilience. Cells with factor loadings greater than 0.35 are highlighted with a bold.

## Study II confirmatory factor analysis

### Method

#### Participants and procedure

Participants completed a 179-item online questionnaire. The questionnaire was published in various groups on Facebook, as the most commonly used social platform. The responses were obtained from the widest possible variations of the groups according to place of residence (e.g., people living in the 2nd district of Budapest, “What I heard in Debrecen,” “What I saw in Sopron”); topic (e.g., nature conservationists, vegetarians); sports (e.g., kayak-canoeists, training plans and experiences, novice runners); profession (e.g., job seekers, social workers); spirituality (e.g., atheist–Christian discussion group, daily spiritual quotes, Christian youth); higher education (e.g., law students, teachers); and others (e.g., mathematics for everyone, motivation for everyday life) in order to reach participants from as many social strata as possible.

Ethical approval for the study was granted by the Research Ethics Committee of the local university (permission number: 70/2019/P/ET/2). Participation was voluntary and anonymous. Informed consent was obtained but no compensation was given. The sample, selected by random sampling, consisted of 1,083 individuals (233 males, 847 females, and three individuals who did not disclose their gender) who completed the 179-item questionnaire online between December 2019 and March 2020. Their sociodemographic characteristics are summarized in Table 6.

**TABLE 6:** Sociodemographic characteristics of the participants of Study II (*n* = 1,083).

Gender	Male: 21.5%	Female: 78.2%	No data: 0.3%		
**Age**	18–25 years old: 33.2%	26–35 years old: 26.2%	36–50 years old: 3.4%	51–65 years old: 8.9%	66–90 years old: 1.3%
**Educational level**	Primary: 2.5%	Secondary: 21.2%	Ongoing higher education: 24.3%	University: 52.0%	
**Profession**	Employee: 66.2%	Student: 22.2%	Entrepreneur: 3.3%	Unemployed: 3.0%	Other: 5.2%
**Monthly income**	Less than 411 EUR: 33.8%	411–822 EUR: 41.7%	More than 822 EUR: 24.5%		
**Subjective financial status**	Below average: 15.4%	Average: 67.1%	Above average: 17.5%		
**Religiosity**	Atheist: 31.1%	Religious in their own way: 47.4%	Religious: 21.5%		
**Importance of religiosity**	Not important at all: 37.0%	Quite important: 32.6%	Very important: 2.1%	It affects all my actions: 1.3%	

Based on the data presented in Table 6, it can be concluded that the Study II sample was slightly less heterogeneous and of slightly different composition than the Study I sample. The majority of the participants in the sample were female (78.2%). On the other hand, there were significantly more young people below the age of 25 (33.1% vs. 2%), while the proportion of those over the age of 50 (1.2% vs. 52.7%) was substantially lower. There were more university students (22.2%) and fewer retired participants (1.4%, as part of the “other” category). As in Study I, the vast majority of participants in Study II (67.1%) also considered their financial status to be average. In terms of religiosity, 68.9% declared themselves to be religious in their own way and 31.1% declared themselves to be atheists, while 3.4% said that religiosity was important (or very important) to them.

#### Measures

Eight of the questions from among all the items were related to sociodemographic data (gender, age, etc., see Table 6). Three questions were specifically related to the subject’s physical and mental well-being: (1) Subjective health status (“Overall, how would you rate your health status?” with response options: 1 = *very bad*, 2 = *bad*, 3 = *average*, 4 = *good*, 5 = *excellent*); (2) Subjective satisfaction with life (“Overall, how satisfied are you with your daily life?” with endpoints for response options: 1 = *completely dissatisfied* and 10 = *completely satisfied*); and (3) Subjective happiness (“All in all, how happy would you say you are?” with endpoints for response options: 1 = *completely unhappy* and 10 = *completely happy*).

##### Mental health test

A detailed description of the measure can be found above and in the Appendix.

##### Shortened aspiration index

The original questionnaire (Kasser and Ryan, 1996) was designed to measure attitudes toward general life goals representing intrinsic (growth, affiliation, and community contribution), extrinsic (wealth, fame, and physical appearance), and health-related motivations. The shortened, 14-item Hungarian version, which contains no reversed items, measures life goals on a seven-point Likert scale (Martos et al., 2006). The Cronbach’s alpha values obtained were 0.80 (Extrinsic Aspiration scale); 0.74 (Intrinsic Aspiration scale); and 0.55 (Health Aspiration scale).

##### Short form of the Beck Depression Inventory

This inventory (Beck et al., 1997; Rózsa et al., 2001) is used to measure the inverse symptoms of mental health. The nine items measure the following symptoms of depression using a four-point Likert scale: social withdrawal, indecisiveness, sleep disorder (parasomnia), fatigue, excessive anxiety about physical symptoms, incapacity, pessimism, lack of satisfaction and joy, and self-blame. For example, the item “I am very worried about physical problems and it’s hard to think of much else” assesses excessive anxiety about physical symptoms. Higher scores obtained by averaging the scales indicate more depressive symptoms. Cronbach’s alpha: 0.86.

##### World Health Organization well-being index

This five-item scale (Bech et al., 1996; Susánszky et al., 2006) provides information about the respondent’s general well-being based on the previous 2-week period using a four-point Likert scale. Higher scores indicate more positive well-being. Cronbach’s alpha: 0.87.

##### Satisfaction with life scale

This scale (Diener et al., 1985; Martos et al., 2014) measures global satisfaction with life using a seven-point Likert scale for five items such as: “If I could live my life over, I would change almost nothing.” A higher score on the scale indicates a higher degree of life satisfaction. Cronbach’s alpha: 0.87.

##### Purpose in life test

Even though the name of the questionnaire (Crumbaugh and Maholick, 1964; Konkolÿ Thege and Martos, 2006) emphasizes life goals only, this 20-item measure assesses life meaning according to Viktor Frankl’s concept, using a seven-point Likert scale. The higher the score, the more the respondent experiences their life as meaningful. Cronbach’s alpha: 0.87.

##### Schema questionnaire–short form

Early maladaptive schemas are thought to be important in connection with mental health as they indicate factors such as Failure to Achieve (“Most people are more talented than I am”) and Emotional Inhibition (“I don’t want people to know about my emotional inhibition personal flaws”). The 95-item questionnaire (Welburn et al., 2002; Unoka et al., 2004) assesses Young’s schemas on six-point Likert scales. The 19 subscales, comprising five items each, are organized into five schema domains: (1) Disconnection/Rejection; (2) Impaired Autonomy/Performance; (3) Other-directedness; (4) Impaired Limits; and (5) Over-vigilance/Inhibition. Subscale scores are obtained by averaging the scale scores. The Cronbach’s alpha values for the subscales were between 0.76 and 0.94.

To summarize, with one exception (the two-item Health subscale of the Shortened Aspiration Index), the scales used in the above tests all had excellent reliability, with Cronbach’s alpha values of above 0.74.

### Results

Confirmatory factor analysis (CFA) was performed with several settings with Mplus (Muthén and Muthén, 1998–2011). The most important results are summarized in Table 7. Row 1 of Table 7 shows the adequacy measures of the EFA model of the MHT. The root mean square error of approximation (RMSEA) (Steiger and Lind, 1980) and standardized root mean square residual (SRMR) (Hu and Bentler, 1999) are absolute fit indices which indicate acceptable fit if their values are less than 0.06 and 0.05 respectively (Maydeu-Olivares et al., 2019). The model has an acceptable fit since the values of the two indicators do not exceed 0.06 (except in one case (RMSEA of Model 1: 0.06), and this is true for all models in Table 7. In the case of RMSEA, the theoretical value should optimally not exceed 0.05 (Steiger and Lind, 1980). A 90% interval estimate is included in the CI_90_(RMSEA) column. This is good if the value 0.05 is included in it (Browne and Cudeck, 1993). The *p*-value of the test that the theoretical value is not greater than 0.05 can be found in the pClose column. This is good if it is greater than 0.05, or, in other words, if it is not significant. Adequacy indicators also include two relative fit indicators, the comparative fit index (CFI) and the Tucker-Lewis Index (TLI). A good fit is indicated when the CFI value reaches 0.95 and the TLI value is not much lower, but definitely higher than 0.90 (Hu and Bentler, 1999). The AIC and BIC are measures of comparative fit and their lower values are favorable (Kenny, 2015).

**TABLE 7:** The main model fit indices in exploratory factor analysis and confirmatory factor analysis of the five-factor model of the MHT.

Chi-square	AIC BIC	RMSEA	CI._90_ (RMSEA)	pClose	CFI TLI	SRMR
**Model 1** Exploratory factor analysis on Study I sample (*n* = 1,736)						
460.963*** (*df* = 61)	80591.0 81186.1	0.061	[0.056; 0.067]	<0.001	0.966 0.924	0.017
**Model 2** Confirmatory factor analysis on Study II sample (*n* = 1, 083)						
497.180*** (*df* = 109)	56140.6 56444.9	0.057	[0.052; 0.063]	0.009	0.934 0.918	0.057
**Model 3** Confirmatory factor analysis on Study II sample (*n* = 1, 083).						
323.529*** (*df* = 107)	55890.6 56204.8	0.043	[0.038; 0.049]	0.980	0.963 0.953	0.043
**Model 4** Second order confirmatory factor analysis on Study II sample (*n* = 1, 083).						
351.044*** (*df* = 112)	55917.9 56207.2	0.044	(0.039; 0.050)	0.960	0.960 0.951	0.048

***p < 0.001. All analyses refer to tests done without item i6R. In case of Model 3 and Model 4, we allow the residual terms of items i3S and i10S to correlate within the Savoring scale, and we allow the residual term of item i9C to correlate with latent factor 1 (Well-being).

In CFA we chose a robust method for model fitting (maximum likelihood mean variance, MLMV), which, in the case of CFA, provides a good alternative to the traditional ML method requiring multidimensional normality (Gao et al., 2020). The CFA results testing Model 2 indicated a high modification index (79) for the residual correlation between i3S and i10S, and also a high modification index (102) for the correlation between i9C and latent factor 1 (Well-being). The latter is in accordance with the corresponding factor loading exceeding the 0.40 level in EFA (see Table 5). In order to improve model fit, we built these correlations in the subsequent CFA models. The improvement is justified by the more favorable fit values in the Model 3 row (RMSEA, SRMR < 0.05; pClose, CFI, TLI > 0.95), having also decreasing AIC and BIC values.

A very important result is that testing our five-factor model on an independent Study II sample, the values obtained indicated good fit for all indicators. Furthermore, when we built in the model a second order factor of the five scales (see row 4), we still had good fit values. To summarize, the five-factor model of the 17-item MHT with the original five scales was confirmed by good fit indices. This result was achieved by allowing one within factor correlation of the residuals (between i3S and i10S), and one cross-loading (between i9C and the latent factor of Well-being) in the final model. In Table 7 all chi-square tests reject the null hypothesis of exact fit, in large samples, like in ours, this often occurs even when the postulated model is only trivially false (Shi et al., 2018).

The internal consistency of all the scales in the MHT was computed with ROPstat (Vargha et al., 2015). Table 8 indicates that the Cronbach’s alpha and McDonald’s omega values mostly being above 0.71 (DeVellis, 2016; Barbera et al., 2020) were adequate for all scales.

**TABLE 8:** Cronbach’s alpha and McDonald’s omega values measuring the internal consistency of the MHT scales in Study II.

Scale	Number of items	Study II (*n* = 1,083)	
		α	ω
Well-being	3	0.899	0.879
Savoring	3	0.718	0.896
Creative and Executive Efficiency	5	0.768	0.894
Self-regulation	3	0.709	0.914
Resilience (without item 6)	3	0.861	0.873

α, Cronbach’s alpha; ω, McDonald’s omega.

## Substantive validity

### Discriminant validity

In order to check the discriminant validity of the MHT scales, we computed for each scale the proportion of variance explained by the other four scales using multivariate linear regression (Lehmann, 1988) on the pooled sample of the two studies (*n* = 2,819). The explained proportions of variance were measured by the appropriate *R*^2^ values. The obtained *R*^2^ values for the Well-being, Savoring, Creative and Executive Efficiency, Self-regulation, and Resilience scales were 0.55, 0.44, 0.41, 0.17, and 0.44 respectively. The unexplained proportions of variance of the five scales were therefore 44.6, 56.4, 58.8, 82.7, and 55.6%, the smallest (44.6%) belonging to Well-being and the largest (82.7%) belonging to Self-regulation. This result, which confirms the discriminant validity of all five scales, shows that each scale has a unique part of at least 44% that is not covered by the other four. Well-being is the only scale where the unexplained proportion of variance is less than 50%, indicating that this scale plays a central role within the five-pillar construct of mental health.

### External and content validity of the mental health test

The content validity of the MHT and the characteristics of the five scales were analyzed with Pearson’s correlations, using different mental health measurement variables from the two studies. Tables 9, 10 indicate these results. Regarding the classification of the strength of the Pearson’s correlations, we followed Cohen’s convention (Cohen, 1988, pp. 79–80). According to this, a correlation is said to be weak if the absolute value of *r* does not reach 0.1, and to be medium, strong, or very strong if the absolute value of *r* reaches 0.3, 0.5, or 0.7 respectively.

**TABLE 9:** Correlations of the MHT subscales with the validating mental health variables of Study I (*n* = 1,736).

Variable	Well-being	Savoring	Creative and Executive Efficiency	Self-regulation	Resilience
Diener	0.728**	0.580**	0.671**	0.344**	0.506**
P-Positive Emotions	0.774**	0.503**	0.520**	0.432**	0.567**
P-Engagement	0.507**	0.386**	0.443**	0.266**	0.372**
P-Positive Relationships	0.585**	0.408**	0.389**	0.258**	0.367**
P-Meaning	0.665**	0.472**	0.548**	0.299**	0.458**
P-Accomplishment	0.580**	0.414**	0.551**	0.279**	0.434**
P-Happiness	0.752**	0.484**	0.460**	0.349**	0.511**
P-Health	0.482**	0.308**	0.338**	0.271**	0.383**
P-Negative Emotions	−0.616**	−0.376**	−0.396**	−0.540**	−0.543**
P-Loneliness	−0.459**	−0.265**	−0.265**	−0.284**	−0.342**
PERMA	0.743**	0.520**	0.579**	0.367**	0.523**
Positive Experience%	0.484**	0.252**	0.262**	0.296**	0.329**
Physical Condition	0.435**	0.265**	0.319**	0.264**	0.340**
General Mental State	0.720**	0.450**	0.469**	0.431**	0.557**
General Health Condition	0.465**	0.292**	0.320**	0.284**	0.372**
Physical Strength	0.448**	0.296**	0.353**	0.240**	0.365**
Worry	−0.345**	−0.148**	−0.154**	−0.304**	−0.357**
Nervous	−0.407**	−0.195**	−0.215**	−0.394**	−0.381**
Tense	−0.422**	−0.203**	−0.215**	−0.384**	−0.390**
Restless	−0.347**	−0.165**	−0.190**	−0.247**	−0.302**
Flow	0.541**	0.521**	0.646**	0.275**	0.429**

*df* = 1,538; *p < 0.05. **p < 0.01. The letter P at the beginning of the variables indicates that it is a subscale of the PERMA-Profiler.

**TABLE 10:** Correlations of the MHT subscales with the validating mental health variables of Study II.

Variable	Well-being	Savoring	Creative and Executive Efficiency	Self-regulation	Resilience
Extrinsic Aspiration	–0.042	0.077*	0.092**	−0.208**	−0.085**
Intrinsic Aspiration	0.168**	0.210**	0.241**	0.040	0.044
Health Aspiration	0.157**	0.218**	0.120**	−0.078*	0.088**
Beck Depression	−0.720**	−0.495**	−0.482**	−0.239**	−0.528**
WHO	0.700**	0.476**	0.439**	0.215**	0.449**
SWLS	0.735**	0.503**	0.449**	0.184**	0.440**
Purpose in Life	0.767**	0.569**	0.607**	0.294**	0.563**
Young Schemas	−0.618**	−0.452**	−0.431**	−0.345**	−0.532**

*df* = 1,083; *p < 0.05. **p < 0.01.

Based on correlations exceeding 0.70 in Tables 9, 10, we can conclude that the three-item Well-being scale measures something very similar as other, traditional tests of well-being such as Diener’s Flourishing Scale, the PERMA-Profiler, the WHO Well-Being Index, and the Satisfaction with Life Scale. The Positive Emotions subscale of the PERMA-Profiler explains the biggest proportion of variance among the subscales. In accordance with the construct content of the Well-being scale, strong correlations were found with the meaning in life variable (Purpose in Life Test) and the Meaning subscale of the PERMA-Profiler, which confirms the significant role of experiencing the meaning of life in terms of well-being. All these results provide ample evidence of the criterion validity of the MHT’s Well-being scale. Convergent validity is indicated by negative emotional states (the Negative Emotions subscale of the PERMA-Profiler, and the “nervous” and “tense” questionnaire items), symptoms of depression (the short form of the Beck Depression Inventory), and maladaptive schemas (Young’s Schema Questionnaire). The significant explanatory power of flow and physical well-being, as well as general health, can also be interpreted as strengthening convergent validity (Carlson and Herdman, 2012).

Regarding the Savoring scale, convergent validity is evidenced by correlations above 0.50 with variables from other MHTs (Diener’s Flourishing Scale, the PERMA-Profiler, Flow, the Satisfaction with Life Scale, the Purpose in Life Test). Convergent validity is further enhanced by moderate, negative correlations with negative emotional states (Beck Depression Inventory and the Negative Emotions subscale of the PERMA-Profiler).

The strong correlation results for the Creative and Executive Efficiency scale with other tests and special questionnaire items of mental health (Diener’s Flourishing Scale, the PERMA-Profiler, the flow items, and the Purpose in Life Test) indicate the fulfillment of convergent validity.

In the case of the convergent validity of the Self-regulation scale, we refer to the mostly medium-level significant correlations with the subscales of the PERMA-Profiler and with general mental state. In line with the meaning of the Self-regulation scale in terms of emotionality and mood control, we can interpret the medium to strong significant correlations with the Negative Emotions scale of the PERMA-Profiler, Young’s Schema Questionnaire, and the “nervous” and the “tense” questionnaire items as contributing to the criterion validity.

As fulfillment of the convergent validity of the Resilience scale, we refer here to its strong correlations with the Diener’s Flourishing Scale, the general mental state questionnaire item, the Purpose in Life Test, and the scales of the PERMA-Profiler, especially with the Positive Emotions and Happiness subscales. Convergent validity is indicated by strong and medium-level negative correlations with negative emotional states (the Negative Emotions subscale of the PERMA-Profiler, and the “nervous” and “tense” questionnaire items), symptoms of depression (Beck Depression Inventory), and maladaptive schemas (Young’s Schema Questionnaire), indicating that a high level of resilience provides some kind of protection against negative emotions and attitudes. This is also confirmed by the medium to strong correlations with scales indicating mental well-being (the Meaning and Accomplishment scales of the PERMA-Profiler, the WHO Well-Being Questionnaire, the flow items, and the Satisfaction with Life Scale). Further evidence is provided by the medium to strong correlation with flow, since certain behavioral competencies are essential for flow, which can be considered as an investment in the individual’s coping system.

### Results with sociodemographic indicators

An examination of the relationship between the five scales of the MHT discussed in our study and the sociodemographic indicators yields many significant results, although these relationships are typically rather weak. For example, for 92% of the correlations examined, Spearman’s *r* is < | 0.20|. A detailed description of these results is beyond the scope of the present paper; thus, we refer to the most striking results only.

**FIGURE 1:**
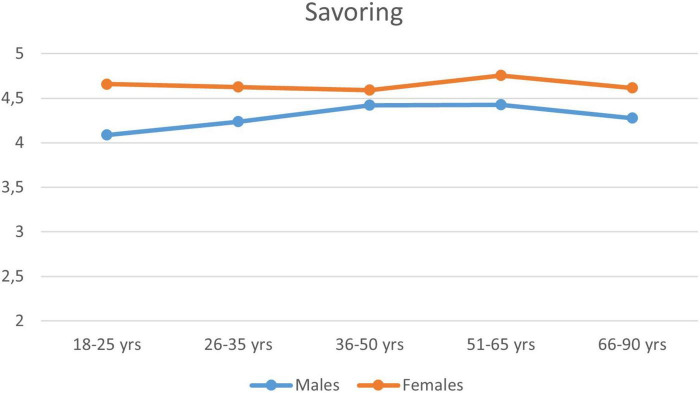
Savoring means in gender by age categories (*n* = 2,817).

The effect of gender and age was analyzed in the pooled sample of the two studies for individuals with valid gender values (*n* = 2,817). Gender means differed significantly in the case of the Savoring scale (males: *M* = 4.33; females: *M* = 4.66), with standard deviations around 1. The gender difference (females giving higher values) was significant in the two-sample *t*-test [*t*(2814) = 6.90; *p* < 0.001], in the Mann–Whitney *U* test (*Z* = 6.50; *p* < 0.001), and in the robust Brunner-Munzel rank test [BM(1128) = 6.58; *p* < 0.001]. Although the Cohen’s *d* (*d* = 0.30, 95% CI [0.216, 0.391]) and eta-squared (η^2^ = 0.017) effect size measures were rather weak, they were already at an interpretable level. Figure 1 shows that the dominance of females was manifested in all age categories.

**FIGURE 2:**
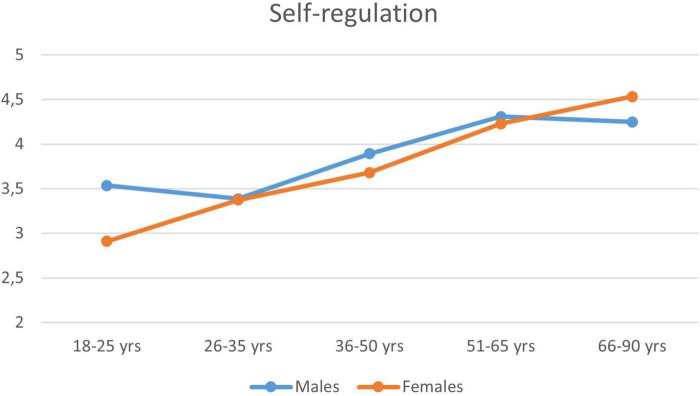
The mean of the Self-regulation scale by gender and age zone (*n* = 2,817).

In the same sample, a more significant effect of age was observed for the Self-regulation (see Figure 1) and Resilience. Using a two-way analysis of variance (ANOVA), the main effect of age was significant for both scales: Self-regulation: *F*(4; 2806) = 53.24; *p* < 0.001; Resilience: *F*(4; 2806) = 32.91; *p* < 0.001, with partial eta-squared effect size values of 0.07 for the former and 0.05 for the latter, apparently stronger than the effect of gender. In the case of self-regulation, the gender × age interaction effect was also significant [*F*(4; 2806) = 4.38; *p* = 0.002]. Figure 2 indicates that it was due to the fact that the level of self-regulation increased linearly with women’s age, while in the case of men it showed an increasing trend only in the 26–50 age range. This was also confirmed by the fact that Spearman’s *r* between self-regulation and age was significantly lower in case of men (*r* = 0.27, *p* < 0.001) than in case of women (*r* = 0.38, *p* < 0.001). The curvilinear effect of Age on Self-Regulation in the male subsample (*n* = 681) was examined also with polynomial regression analysis, where powers of standardized age were entered consecutively into the regression model up to power 5. The last power that significantly increased the *R*-square value was the cubic term [*R*^2^ = 0.08; *R*^2^_increase = 0.01; *F*_*increase*_(1,677) = 9.13, *p* = 0.003]. This cubic curvilinear effect of Age on Self-Regulation in the male subsample is, however, negligible relative to the substantially stronger linear relationship that can be seen in the female subsample (*n* = 2,135), where no power increased significantly the linear effect of age (*R*^2^ = 0.14).

The effects of the other sociodemographic variables were assessed separately in the two studies. Regarding level of education, the strongest positive correlation was with the Creative and Executive Efficiency scale (*r* = 0.16 in sample I, *r* = 0.13 in sample II, *p* < 0.001). Subjective financial status showed a stronger relationship in both samples. In Sample I, subjective financial status was positively related to Well-being, with *r* = 0.21, and to Creative and Executive Efficiency, with *r* = 0.12 (both significant at *p* < 0.001 level). However, in Sample II subjective financial status also had a significant positive relationship with Well-being (*r* = 0.23), Resilience (*r* = 0.20), and Creative and Executive Efficiency (*r* = 0.17), all at *p* < 0.001 level. A slightly stronger but similar pattern was obtained in this sample for correlations with monthly income (Well-being: *r* = 0.17, Resilience: *r* = 0.27, Creative and Executive Efficiency: *r* = 0.25). Financial status therefore is positively related to well-being, resilience, and coping with difficult situations. Finally, the importance of religion in Sample II had a weak but significant (*p* < 0.001) positive relationship with the Well-being (*r* = 0.14) and Savoring (*r* = 0.13) scales.

### Temporal stability of the mental health test

In Study I, participants could voluntarily provide their email addresses for a possible replication of the online investigation. Participants who provided this information were asked to fill in the same questionnaire 2 weeks (Time 2) and 11 months (Time 3) after the first investigation (Time 1). This enabled us to compute test–retest correlations to measure the temporal stability of the MHT scales. In major categories of financial status (below average, average, above average) the differences among Time 1, Time 2, and Time 3 percentages were never greater than 2 percentage points. Regarding age the Time 1 mean of Time 2 retention sample (52.3 years) differed only slightly from that of Time 1 sample (51.3 years). Those Ss filling in the questionnaires both at Time 1 and at Time 3 were slightly older at Time 1 than those who filled in the questionnaires only at Time 1 (54.4 vs. 50.6 years; *t*(1734) = 5.18, *p* < 0.001; Cohen’s *d* = 0.33, 95% CI [0.203, 0.451]).

At Time 2, we had 648 usable cases and Cronbach’s alpha values varied between 0.79 and 0.87, whereas at Time 3 we had 304 usable cases and Cronbach’s alpha values varied between 0.79 and 0.90. It can therefore be concluded that the internal consistency of the MHT scales shows excellent temporal stability for all scales at Time 2 and Time 3. The test–retest correlations between the Time 1 and Time 2, and between the Time 1 and Time3 MHT scale values are summarized in Table 11 and reflect an excellent level of temporal stability for all scales at Time 2, and a good level of temporal stability at Time 3, almost 1 year after the initial investigation.

**TABLE 11:** Test–retest correlations of the MHT scales in Study I for replication after 2 weeks (Time 2; *n* = 581) and 11 months (Time 3; *n* = 270).

Scale	Time 1 vs. Time 2	Time 1 vs. Time 3
Well-being	0.762	0.642
Savoring	0.774	0.623
Creative and Executive Efficiency	0.799	0.652
Self-regulation	0.838	0.709
Resilience	0.784	0.697
MHT Total	0.882	0.755

*df* = 579; 268; *p < 0.05, **p < 0.01.

## Discussion

The objective of this paper was to conceptualize Maintainable Positive Mental Health Theory and to develop and validate the MHT that operationalizes this model. The theoretical basis of the MPMHT is a mental health construct in line with the mental health definition of the WHO (Galderisi et al., 2015). MPMHT emphasizes that the measurement of mental health must go beyond operationalizations that define the concept in terms of observable characteristics of well-being (e.g., Butler and Kern, 2016; Lukat et al., 2016), or characteristics that are listed as mirror opposites of mental disorders (e.g., Huppert and So, 2013; Caprara et al., 2019; Oláh, 2019).

Our results support the conceptual definition of MPMHT that refers to a degree of global well-being that goes hand in hand with good emotional, psychological, social, and spiritual functioning, resilience, coping, and savoring capacity, as well as mental health sustainability on an ongoing basis, with development and flexible adaptation to changing conditions guaranteed by competencies and personality factors.

Although various psychological tests exist, with different positive psychological constructs (see Sections “Introduction,” “Measures,” and “Measures”), the MHT is the first test to have a five-dimensional complex structure (Well-being, Savoring, Creative and Executive Efficiency, Self-regulation, and Resilience), with the aim of covering the wide spectrum of mental health.

The most important finding in our online cross-sectional studies is that the five-dimensional structural validity of the 17-item MHT was verified by EFA using a large sample (Study I, *n* = 1,736). In turn, using CFA, the five-factor MHT model with the original five scales was confirmed by excellent fit indices in another large and independent sample (Study II, *n* = 1,083). In addition, the internal consistency and temporal stability of the scales was proven. By analyzing the discriminant validity using multivariate linear regression, we were able to conclude that all five scales have a significant individual part of at least 44% that is not covered by the other four scales. The scale with the highest unique variance of 82.7% is Self-regulation, although the unique variances of the Savoring and Creative and Executive Efficiency scales also exceed 55%. The Well-being scale is the only one for which the unexplained proportion of variance is less than 50%. This suggests that, among the five components of mental health, well-being plays a central role. To expand and confirm the individual meaning of the scales, several tests and individual questionnaire items were used in correlation analyses, and sociodemographic variables in ANOVAs.

The results support that the Well-being scale confidently measures subjective well-being, which itself comprises several components (biological, psychological, social, and spiritual). The Well-being scale correlates at a high level with related tests such as Diener’s Flourishing Scale, the PERMA-Profiler, the WHO Well-Being Questionnaire, the Satisfaction with Life Scale, and the Purpose in Life Test, thus it can be considered as a very similar test. An important result is that the existence of meaningful goals and the absence of depressive symptoms and maladaptive schemas are an integral part of the construct measured by the Well-being scale (see Table 10). Well-being is related to the individual’s subjective financial status, although this relationship is weak. Well-being has the strongest relationship with the other components of mental health.

The Savoring scale measures how individuals are able to mentally mobilize their previous positive, joyful memories and experiences to generate mental well-being, reliving them in the present and, furthermore, extending them to future events. This ability appears to be more prevalent among women of all ages than men (see Figure 1). The results of the validity studies show that the Savoring scale is closely related to the main indicators of all the MHTs involved: Diener’s Flourishing Scale, the PERMA-Profiler, Flow, the WHO Well-Being Questionnaire, the Satisfaction with Life Scale, and the Purpose in Life Test (see Tables 9, 10), which confirms the validity of its construct. The Creative and Executive Efficiency scale measures how individuals are able to cope with the difficulties they encounter by mobilizing their various competencies in the difficult, stressful, and challenging situations of life. Furthermore, it measures how individuals are able to provide successful individual and social problem-solving behavior. The Creative and Executive Efficiency scale shows strong correlations with other tests of mental health (the Flourishing Scale, the PERMA-Profiler, the Purpose in Life Test), which indicates the fulfillment of convergent validity (see Tables 9, 10). It is also related to the competence component, in that a person who achieves a high value on this scale also has a better chance of reaching flow experience (see Table 9). Further results of the validity studies show that the Creative and Executive Efficiency scale is a negative predictor of negative emotions, symptoms of depression, and maladaptive schemas.

The Self-regulation scale provides information about a person’s ability to regulate and control emotions, temperament, and negative states. Positive correlations between the Self-regulation scale and general mental state, and negative correlations with negative emotional states were consistently obtained, indicating the general good functioning of the individual (see Tables 9, 10). The construct measured by the Self-regulation scale correlates weakly with age. Self-regulation is sometimes more successful in the case of older people, and this positive age effect is more dominant in women (see Figure 2).

The Resilience scale measures the level of mental capacities and resources that can be mobilized when a person faces unexpected, stressful, and difficult situations. The higher the level of resilience, the more quickly the individual is able to recover from a sudden, unexpected stressful situation. Our results confirm that a good capacity to experience flow, meaningful life goals, lack of negative emotional states, satisfaction with life, and the absence of depressive symptoms and maladaptive schemas contribute to a higher level of resilience (see Tables 9, 10). Like self-regulation, resilience has a positive but weak relationship with age.

One of the key findings of our study is that there are competencies behind the different components of mental health. This result implies that these competences could be trained, improved, and strengthened by their nature. This is most obvious in the case of creative and executive efficiency, although, based on the positive correlation with age, it must also be true of self-regulation and resilience. Savoring also implies mental ability that can be improved with cognitive techniques. As a result, the level of experienced subjective well-being, satisfaction with life, and, in ordinary terms, happiness can be significantly increased. Although, this positive prognosis can be confirmed by subsequent research which aim to demonstrate how these competencies can be developed.

One limitation of our studies is the online recruitment of participants: despite the large size of the samples, they cannot be considered representative. The recruitment technique may greatly influence several aspects (e.g., age distribution; see Tables 2, 6). Our analyses are based solely on data from verbal questionnaires, while the MHT scales are based, among other things, on specific behaviors, mental operations, attitudes, and so on. For this reason, to confirm empirical validity it would also be very important to verify the validity of the scales with other types of psychological variables (e.g., rating scales, direct observations, clinical symptoms, specific data measuring physical condition, sociometric ranking, etc.). By way of example, it would be useful to compare mental health as measured by the MHT with the variables of 360-degree studies (Mahar and Strobert, 2010). The meaning of the scales should also be confirmed by examining various clinical cases with identified psychiatric diagnoses.

## Conclusion

In sum, we believe our findings show in its present, 17-item form, designed for adults, the MHT can provide a comprehensive picture of mental health in terms of MPMHT. The MHT has a number of advantages over existing measures of well-being and mental health, making it a preferable measurement device for use in future research. Applying the MPMHT approach in consultations, in skill-improvement sessions, in relationship training, in group sessions, and in behavioral therapy could help to improve the level of mental health in psychologically healthy people. Future research should examine the MHT in the clinical population and apply MPMHT in positive clinical psychology.

## Data availability statement

The raw data supporting the conclusions of this article will be made available by the authors, without undue reservation.

## Ethics statement

The studies involving human participants were reviewed and approved by Research Ethics Committee of the Faculty of Education and Psychology of ELTE (permission number: 2019/61) and the Research Ethics Committee of the Institute of Psychology of Károli Gáspár University (permission number: 70/2019/P/ET/2). The patients/participants provided their written informed consent to participate in this study.

## Author contributions

All authors listed have made a substantial, direct, and intellectual contribution to the work, and approved it for publication.

## Footnotes

1 Seniors Group; Through Engineers’ Eyes; Professionals Group; Danube Anglers; Youth of Budapest; Soccer fans; We enjoy cooking; Gardeners; Classical music lovers; Viticulturists and Winemakers Club.

2 Their place of residence could be identified based on the name of the settlement provided by them.

3
https://dailynewshungary.com/happiness-map-of-hungary/

## Appendix

**The Mental Health Test (MHT)**

The following statements are designed to provide information about your perceptions of wellness. Please consider each statement carefully and thoughtfully, then enter an X to indicate the response option with which you most agree. There are no right or wrong answers.

**Table:**

	Strongly disagree	Disagree	Slightly disagree	Slightly agree	Agree	Strongly agree
(1) Joy is present more than sorrow in my everyday life./W						
(2) I easily become impatient./SR						
(3) It’s easy for me to revive the joy from pleasant memories./S						
(4) I tend to bounce back quickly after hard times./R						
(5) I often have ideas that are taken further by others./C						
(6) I have a hard time making it through stressful events./R						
(7) Others describe me as a problem solver./C						
(8) I am impulsive: I act first and think second./SR						
(9) I can successfully achieve targets which I set for myself./C						
(10) I like to store memories of fun times that I go through so that I can recall them later./S						
(11) It does not take me long to recover from a stressful event./R						
(12) I can make myself feel good by imagining what a happy time that is about to happen will be like./S						
(13) I tend to take a long time to get over set-backs in my life./R						
(14) My general psychological state is good./W						
(15) I am good at work that needs new and original ideas./C						
(16) I become frustrated when something does not happen the way I planned it./SR						
(17) I often know what people are thinking and feeling./C						
(18) How do you feel about your life as a whole?/W (1: Very bad, 6: Very good)						

**Scaling guide**

Well-being (W): The average of the scores for items 1, 14, and 18.

Savoring (S): The average of the scores for items 3, 10, and 12.

Creative and Executive Efficiency (C): The average of the scores for items 5, 7, 9, 15, and 17.

Self-regulation (SR): The average of the scores for items 2, 8, and 16, after inverting all three items with the 7 – x transformation (x is the original, 7 – x is the inverted score).

Resilience (R): The average of the scores for items 4, 11, and 13, after inverting item 13 with the 7 – x transformation (x is the original, 7 – x is the inverted score).
