# Corrigendum: A new complex mental health test in a positive psychological framework

**DOI:** 10.3389/fpsyg.2023.1290998

**Published:** 2023-10-26

**Authors:** Virág Zábó, Attila Oláh, András Vargha

**Affiliations:** ^1^Doctoral School of Psychology, ELTE Eötvös Loránd University, Budapest, Hungary; ^2^Institute of Psychology, ELTE Eötvös Loránd University, Budapest, Hungary; ^3^Institute of Research on Adult Education and Knowledge Management, ELTE Eötvös Loránd University, Budapest, Hungary; ^4^Person- and Family-oriented Health Science Research Group, Institute of Psychology, Faculty of Humanities and Social Sciences, Károli Gáspár University of the Reformed Church in Hungary, Budapest, Hungary

**Keywords:** happiness, subjective well-being, mental health, mental health test, positive psychology (PP1.0 and PP2.0), positive psychological assessments, maintainable positive mental health theory

In the published article, there were errors in affiliations 1 and 2. Instead of

“^1^Positive Psychology Research Group, Institute of Psychology, Faculty of Education and Psychology, Eötvös Loránd University, Budapest, Hungary” and

^“2^Doctoral School of Psychology, ELTE Eötvös Loránd University, Budapest, Hungary”,

it should be

“^1^Doctoral School of Psychology, ELTE Eötvös Loránd University, Budapest, Hungary” and

“^2^Institute of Psychology, ELTE Eötvös Loránd University, Budapest, Hungary”.

Attila Oláh should be affiliated to “Institute of Psychology, ELTE Eötvös Loránd University, Budapest, Hungary”.

András Vargha should be affiliated to “Institute of Psychology, ELTE Eötvös Loránd University, Budapest, Hungary” and “Person- and Family-oriented Health Science Research Group, Institute of Psychology, Faculty of Humanities and Social Sciences, Károli Gáspár University of the Reformed Church in Hungary, Budapest, Hungary”.

Additionally, in the published article, there was an error in the Appendix/The Mental Health Test (MHT). Item 16 of the questionnaire was incorrectly written as “I easily become impatient./SR”. The corrected Item 16 appears below.

(16) I become frustrated when something does not happen the way I planned it./SR

The authors apologize for these errors and state that they do not change the scientific conclusions of the article in any way. The original article has been updated.

